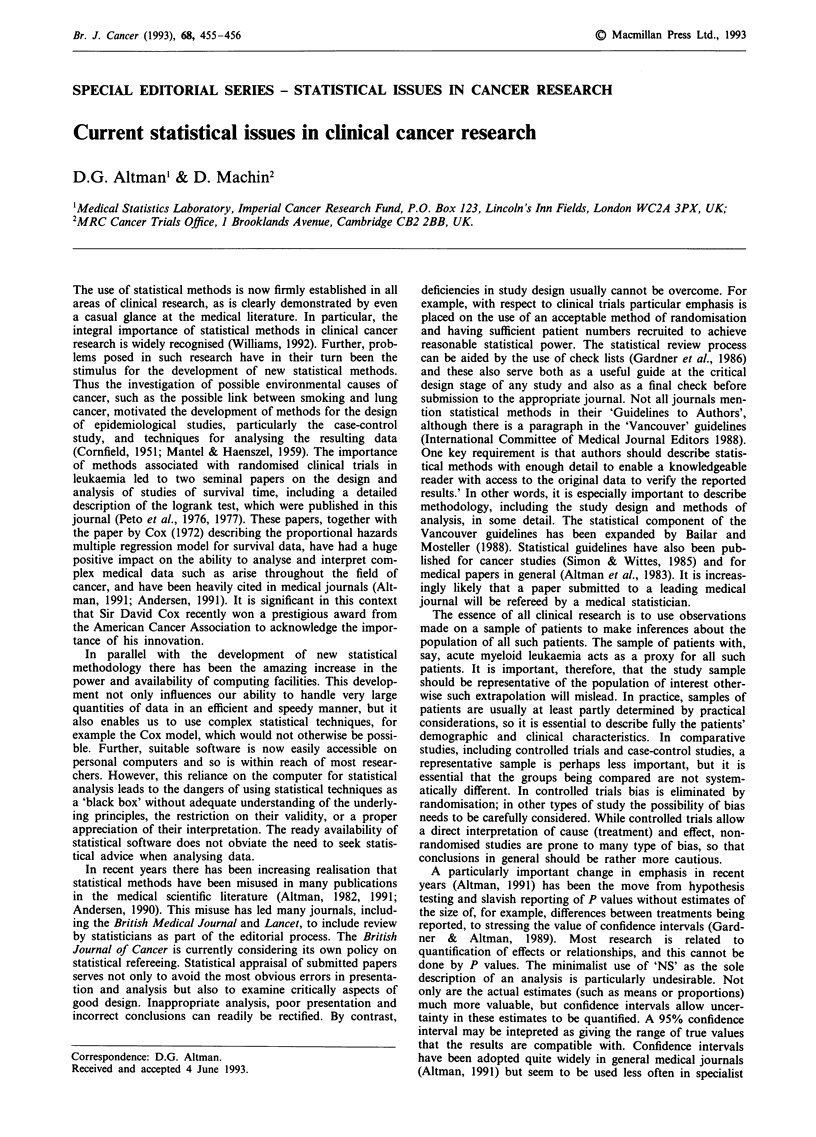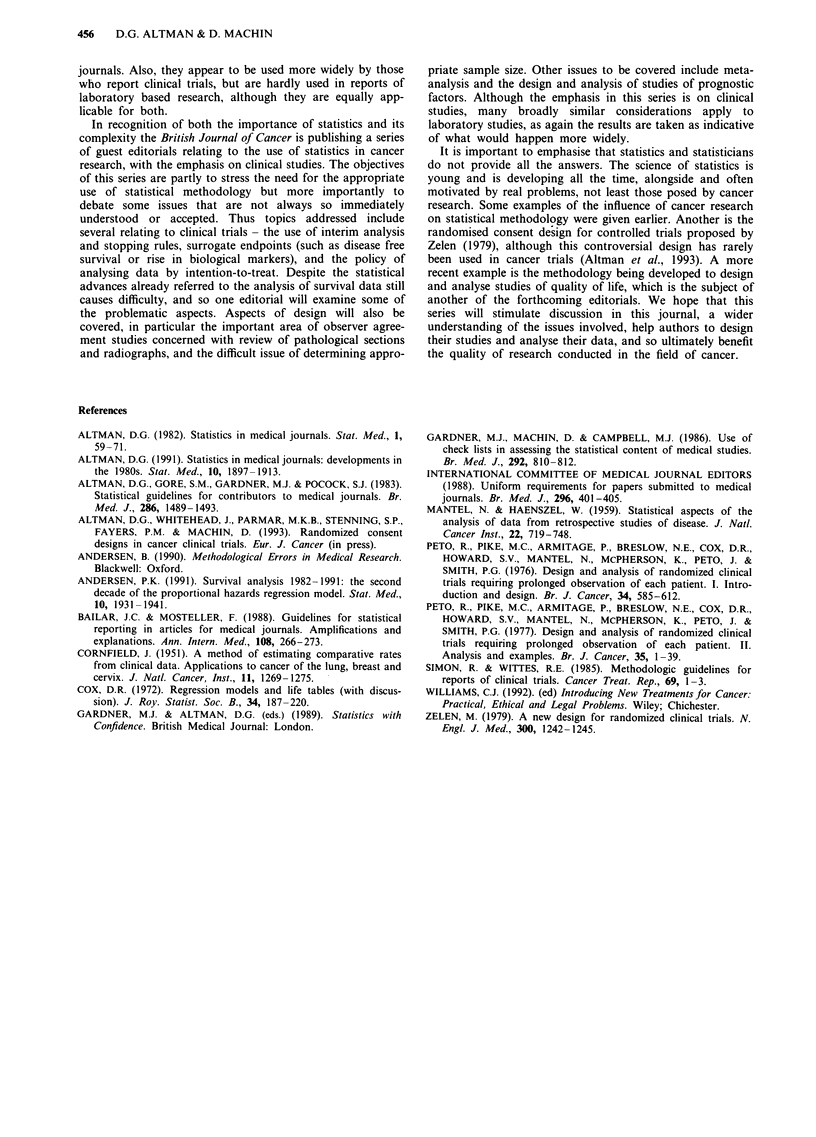# Current statistical issues in clinical cancer research.

**DOI:** 10.1038/bjc.1993.368

**Published:** 1993-09

**Authors:** D. G. Altman, D. Machin


Br. J. Cancer (1993), 68, 455–456 © Macmillan Press Ltd., 1993

SPECIAL EDITORIAL SERIES – STATISTICAL ISSUES IN CANCER RESEARCH

### Current statistical issues in clinical cancer research

D.G. Altman[1] & D. Machin[2]

[1]*Medical Statistics Laboratory, Imperial Cancer Research Fund, P.O. Box 123, Lincoln's Inn Fields, London WC2A 3PX, UK;* [2]*MRC Cancer Trials Office, 1 Brooklands Avenue, Cambridge CB2 2BB, UK.*

The use of statistical methods is now firmly established in all areas of clinical research, as is clearly demonstrated by even a casual glance at the medical literature. In particular, the integral importance of statistical methods in clinical cancer research is widely recognised (Williams, 1992). Further, problems posed in such research have in their turn been the stimulus for the development of new statistical methods. Thus the investigation of possible environmental causes of cancer, such as the possible link between smoking and lung cancer, motivated the development of methods for the design of epidemiological studies, particularly the case-control study, and techniques for analysing the resulting data (Cornfield, 1951; Mantel & Haenszel, 1959). The importance of methods associated with randomised clinical trials in leukaemia led to two seminal papers on the design and analysis of studies of survival time, including a detailed description of the logrank test, which were published in this journal (Peto *et al.*, 1976, 1977). These papers, together with the paper by Cox (1972) describing the proportional hazards multiple regression model for survival data, have had a huge positive impact on the ability to analyse and interpret complex medical data such as arise throughout the field of cancer, and have been heavily cited in medical journals (Altman, 1991; Andersen, 1991). It is significant in this context that Sir David Cox recently won a prestigious award from the American Cancer Association to acknowledge the importance of his innovation.

In parallel with the development of new statistical methodology there has been the amazing increase in the power and availability of computing facilities. This development not only influences our ability to handle very large quantities of data in an efficient and speedy manner, but it also enables us to use complex statistical techniques, for example the Cox model, which would not otherwise be possible. Further, suitable software is now easily accessible on personal computers and so is within reach of most researchers. However, this reliance on the computer for statistical analysis leads to the dangers of using statistical techniques as a 'black box' without adequate understanding of the underlying principles, the restriction on their validity, or a proper appreciation of their interpretation. The ready availability of statistical software does not obviate the need to seek statistical advice when analysing data.

In recent years there has been increasing realisation that statistical methods have been misused in many publications in the medical scientific literature (Altman, 1982, 1991; Andersen, 1990). This misuse has led many journals, including the *British Medical Journal* and *Lancet*, to include review by statisticians as part of the editorial process. The *British Journal of Cancer* is currently considering its own policy on statistical refereeing. Statistical appraisal of submitted papers serves not only to avoid the most obvious errors in presentation and analysis but also to examine critically aspects of good design. Inappropriate analysis, poor presentation and incorrect conclusions can readily be rectified. By contrast, deficiencies in study design usually cannot be overcome. For example, with respect to clinical trials particular emphasis is placed on the use of an acceptable method of randomisation and having sufficient patient numbers recruited to achieve reasonable statistical power. The statistical review process can be aided by the use of check lists (Gardner *et al.*, 1986) and these also serve both as a useful guide at the critical design stage of any study and also as a final check before submission to the appropriate journal. Not all journals mention statistical methods in their 'Guidelines to Authors', although there is a paragraph in the 'Vancouver' guidelines (International Committee of Medical Journal Editors 1988). One key requirement is that authors should describe statistical methods with enough detail to enable a knowledgeable reader with access to the original data to verify the reported results.' In other words, it is especially important to describe methodology, including the study design and methods of analysis, in some detail. The statistical component of the Vancouver guidelines has been expanded by Bailar and Mosteller (1988). Statistical guidelines have also been published for cancer studies (Simon & Wittes, 1985) and for medical papers in general (Altman *et al.*, 1983). It is increasingly likely that a paper submitted to a leading medical journal will be refereed by a medical statistician.

The essence of all clinical research is to use observations made on a sample of patients to make inferences about the population of all such patients. The sample of patients with, say, acute myeloid leukaemia acts as a proxy for all such patients. It is important, therefore, that the study sample should be representative of the population of interest otherwise such extrapolation will mislead. In practice, samples of patients are usually at least partly determined by practical considerations, so it is essential to describe fully the patients' demographic and clinical characteristics. In comparative studies, including controlled trials and case-control studies, a representative sample is perhaps less important, but it is essential that the groups being compared are not systematically different. In controlled trials bias is eliminated by randomisation; in other types of study the possibility of bias needs to be carefully considered. While controlled trials allow a direct interpretation of cause (treatment) and effect, non-randomised studies are prone to many type of bias, so that conclusions in general should be rather more cautious.

A particularly important change in emphasis in recent years (Altman, 1991) has been the move from hypothesis testing and slavish reporting of $P$ values without estimates of the size of, for example, differences between treatments being reported, to stressing the value of confidence intervals (Gardner & Altman, 1989). Most research is related to quantification of effects or relationships, and this cannot be done by $P$ values. The minimalist use of 'NS' as the sole description of an analysis is particularly undesirable. Not only are the actual estimates (such as means or proportions) much more valuable, but confidence intervals allow uncertainty in these estimates to be quantified. A 95% confidence interval may be intepreted as giving the range of true values that the results are compatible with. Confidence intervals have been adopted quite widely in general medical journals (Altman, 1991) but seem to be used less often in specialist

Correspondence: D.G. Altman.
Received and accepted 4 June 1993.

journals. Also, they appear to be used more widely by those who report clinical trials, but are hardly used in reports of laboratory based research, although they are equally applicable for both.

In recognition of both the importance of statistics and its complexity the *British Journal of Cancer* is publishing a series of guest editorials relating to the use of statistics in cancer research, with the emphasis on clinical studies. The objectives of this series are partly to stress the need for the appropriate use of statistical methodology but more importantly to debate some issues that are not always so immediately understood or accepted. Thus topics addressed include several relating to clinical trials – the use of interim analysis and stopping rules, surrogate endpoints (such as disease free survival or rise in biological markers), and the policy of analysing data by intention-to-treat. Despite the statistical advances already referred to the analysis of survival data still causes difficulty, and so one editorial will examine some of the problematic aspects. Aspects of design will also be covered, in particular the important area of observer agreement studies concerned with review of pathological sections and radiographs, and the difficult issue of determining appropriate sample size. Other issues to be covered include meta-analysis and the design and analysis of studies of prognostic factors. Although the emphasis in this series is on clinical studies, many broadly similar considerations apply to laboratory studies, as again the results are taken as indicative of what would happen more widely.

It is important to emphasise that statistics and statisticians do not provide all the answers. The science of statistics is young and is developing all the time, alongside and often motivated by real problems, not least those posed by cancer research. Some examples of the influence of cancer research on statistical methodology were given earlier. Another is the randomised consent design for controlled trials proposed by Zelen (1979), although this controversial design has rarely been used in cancer trials (Altman *et al.*, 1993). A more recent example is the methodology being developed to design and analyse studies of quality of life, which is the subject of another of the forthcoming editorials. We hope that this series will stimulate discussion in this journal, a wider understanding of the issues involved, help authors to design their studies and analyse their data, and so ultimately benefit the quality of research conducted in the field of cancer.

### References

ALTMAN, D.G. (1982). Statistics in medical journals. *Stat. Med.*, **1**, 59–71.

ALTMAN, D.G. (1991). Statistics in medical journals: developments in the 1980s. *Stat. Med.*, **10**, 1897–1913.

ALTMAN, D.G., GORE, S.M., GARDNER, M.J. & POCOCK, S.J. (1983). Statistical guidelines for contributors to medical journals. *Br. Med. J.*, **286**, 1489–1493.

ALTMAN, D.G., WHITEHEAD, J., PARMAR, M.K.B., STENNING, S.P., FAYERS, P.M. & MACHIN, D. (1993). Randomized consent designs in cancer clinical trials. *Eur. J. Cancer* (in press).

ANDERSEN, B. (1990). *Methodological Errors in Medical Research.* Blackwell: Oxford.

ANDERSEN, P.K. (1991). Survival analysis 1982–1991: the second decade of the proportional hazards regression model. *Stat. Med.*, **10**, 1931–1941.

BAILAR, J.C. & MOSTELLER, F. (1988). Guidelines for statistical reporting in articles for medical journals. Amplifications and explanations. *Ann. Intern. Med.*, **108**, 266–273.

CORNFIELD, J. (1951). A method of estimating comparative rates from clinical data. Applications to cancer of the lung, breast and cervix. *J. Natl. Cancer, Inst.*, **11**, 1269–1275.

COX, D.R. (1972). Regression models and life tables (with discussion). *J. Roy. Statist. Soc. B.*, **34**, 187–220.

GARDNER, M.J. & ALTMAN, D.G. (eds.) (1989). *Statistics with Confidence.* British Medical Journal: London.

GARDNER, M.J., MACHIN, D. & CAMPBELL, M.J. (1986). Use of check lists in assessing the statistical content of medical studies. *Br. Med. J.*, **292**, 810–812.

INTERNATIONAL COMMITTEE OF MEDICAL JOURNAL EDITORS (1988). Uniform requirements for papers submitted to medical journals. *Br. Med. J.*, **296**, 401–405.

MANTEL, N. & HAENSZEL, W. (1959). Statistical aspects of the analysis of data from retrospective studies of disease. *J. Natl. Cancer Inst.*, **22**, 719–748.

PETO, R., PIKE, M.C., ARMITAGE, P., BRESLOW, N.E., COX, D.R., HOWARD, S.V., MANTEL, N., MCPHERSON, K., PETO, J. & SMITH, P.G. (1976). Design and analysis of randomized clinical trials requiring prolonged observation of each patient. I. Introduction and design. *Br. J. Cancer*, **34**, 585–612.

PETO, R., PIKE, M.C., ARMITAGE, P., BRESLOW, N.E., COX, D.R., HOWARD, S.V., MANTEL, N., MCPHERSON, K., PETO, J. & SMITH, P.G. (1977). Design and analysis of randomized clinical trials requiring prolonged observation of each patient. II. Analysis and examples. *Br. J. Cancer*, **35**, 1–39.

SIMON, R. & WITTES, R.E. (1985). Methodologic guidelines for reports of clinical trials. *Cancer Treat. Rep.*, **69**, 1–3.

WILLIAMS, C.J. (1992). (ed) *Introducing New Treatments for Cancer: Practical, Ethical and Legal Problems.* Wiley; Chichester.

ZELEN, M. (1979). A new design for randomized clinical trials. *N. Engl. J. Med.*, **300**, 1242–1245.